# Statistical points and pitfalls: growth modeling

**DOI:** 10.1007/s40037-022-00703-1

**Published:** 2022-03-16

**Authors:** Christy K. Boscardin, Stefanie S. Sebok-Syer, Martin V. Pusic

**Affiliations:** 1 Department of Medicine and Anesthesia, University of California San Francisco, San Francisco, USA; 2 Department of Emergency Medicine, Stanford University, Palo Alto, CA USA; 3 Department of Pediatrics, Harvard University, Boston, MA USA

The overall purpose of the ‘Statistical Points and Pitfalls’ series is to help readers and researchers alike increase awareness of how to use statistics and why/how we fall into inappropriate choices or interpretations. We hope to help readers understand common misconceptions and give clear guidance on how to avoid common pitfalls by offering simple tips to improve your reporting of quantitative research findings. Each entry discusses a commonly encountered inappropriate practice and alternatives from a pragmatic perspective with minimal mathematics involved. We encourage readers to share comments on or suggestions for this section on Twitter, using the hashtag: #mededstats

In this entry, we provide an overview of a longitudinal data analytic technique, *growth modeling*, that is gaining popularity in health professions education. Our purpose is to provide a brief explanation of the method and key points to consider for critical appraisal of its use.

## What is growth modeling?

Many educational research questions require investigation of change, development, or growth over time using repeated measures, including early identification of struggling learners and improved precision in the timing of interventions. The primary purpose of longitudinal data analysis, also known as growth modeling, is to understand and characterize changes in an assessment measure over time. A frequent example is the modeling of learning trajectories where participants improve their performance as they spend time learning. Growth modeling has advantages over previous methods such as Repeated Measures ANOVA in that it can take into account clustering, and provides flexibility with non-continuous dependent variables, as well as being more tolerant of missing data [1].

Using growth modeling, researchers can address questions related to: a) descriptions of change or growth (absolute or relative magnitude of change over time for an individual or group)—*e.g. Does empathy decrease over time during medical school?* b) prediction of growth (models to predict the future status of an individual or group given current and past information)—e.g. *Does STEP 1 score predict resident milestones development? *c) added value (providing explanations for the causes of growth by associating change with other explanatory variables)—e.g. *What individual and learning environment characteristics influence increase in wellbeing? *Both structural equation modeling (SEM) [2, 3] and multi-level modeling (MLM) [4, 5] are common frameworks for conducting growth modeling.

## Example study

Suppose you are interested in learners’ acquisition of medical knowledge throughout medical school training and the factors associated with this longitudinal growth. This information can guide curricular interventions for learners needing additional support. In the following example, students completed three progress tests assessing their acquisition of basic medical knowledge spanning the first two years of the curriculum. The medical knowledge assessments were administered at 0 months (Time 1), 7 months (Time 2), and 16 months (Time 3). The time gap between the first two test occasions (time 1 and time 2) is 7 months, but the third test occasion (time 3) is longer with 9 months since time 2.

## What are the key statistical points and pitfalls to avoid?

To optimize utility, make sound inference, and appropriately report the results of growth modeling, we need to consider several issues including: a) data requirements, b) model fit, and c) inclusion of explanatory variables.

### Data requirements and pitfalls of small sample size

**Fig. 1:**
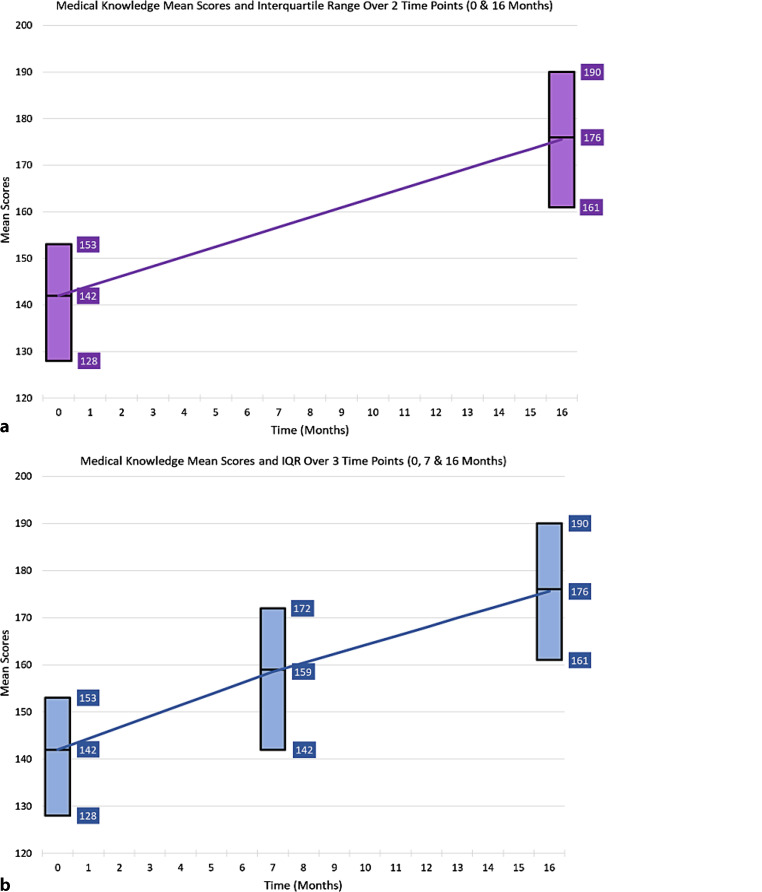
Comparison of growth trajectories at 2 time points and 3 time points (**a** 2 time / **b** 3 time). Addition of the middle point allows better interpretation of the rate of growth in medical knowledge (see text)

To use growth modeling, data from at least three time points are required in a longitudinal study design. With only two time points (pre/post data), the information is limited to change (gain) rather than providing additional information such as the shape of the growth curve (linear, non-linear), timing of change, or power and precision for studying growth. As illustrated in Fig. 1, students in the example study increased in their performance by about 17 points between the three time points: time 1 (*m* [mean] = 142, *SD* [Standard Deviation] = 23), time 2 (*m* = 159, *SD* = 26), time 3 (*m* = 176, *SD* = 24). However, without the inclusion of time 2, it would be difficult to compare the change between time periods given that there was a longer lag between time 2 and time 3. Despite the increased lag time between time 2 and time 3, the mean growth during time 1 to 2 and time 2 to 3 were the same (17 points) illustrating a slower growth between time 2 and 3 as illustrated in Fig. 1. Further investigation also revealed that the standard deviation increased over time. This information is valuable for curricular evaluation as well as targeting interventions.

Sample size requirements will depend on the complexity of the data and the amount of variance explained by the model; however, a minimum sample size of around *n* = 100 is commonly recommended based on simulation studies to reliably estimate growth models [1, 6]. One of the most common pitfalls that we observe with health professions education studies using growth modeling is related to inadequate sample size. Simulated and empirical studies have demonstrated the potential problems with small sample size, especially with non-normal distribution or missing data, including bias in estimates and susceptibility to Type 1 error. Given that sample size adequacy will vary depending on data characteristics, we recommend checking for model fit in addition to stability of the estimated parameters for final determination. Bayesian estimation has been used as an alternative approach to address some of the issues around model identification typically associated with small sample size. Additionally, sample size and power calculations for specific settings can be performed using Monte Carlo methods in statistical packages (e.g. Mplus) [7].

### Determining model fit

Another pitfall associated with reporting growth modeling is the lack of transparency around model evaluation and selection of a final model. Depending on the analytic framework (SEM or MLM), model fit indices can provide information about validity of your models and justification for a selected model. As a rule of thumb for SEM, Root Mean Square Error of Approximation (RMSEA) smaller than 0.06 and a Comparative Fit Index (CFI) and Tucker Lewis Index (TLI) larger than 0.95 indicate relatively good fit [8]. The Bayesian Information Criteria (BIC) or the Akaike’s Information Criteria (AIC) to rank order models have also been recommended for model fit comparison with lower values indicating better fit [9]. For MLM, similar to other regression models, *R*^*2*^ is often used to reflect the fit of the model. This can be a useful index when you have covariates and predictors in the model (e.g. the effect of self-regulation on performance over time). Both SEM and MLM model fit indices should be considered with caution given the lack of consensus around cut-offs for goodness of fit. In our example study with three time points, the model yielded CFI = 0.99, TLI = 0.98, and RMSEA = 0.06, suggesting an adequate model fit.

### Explanatory variables to aid in interpretation

**Fig. 2:**
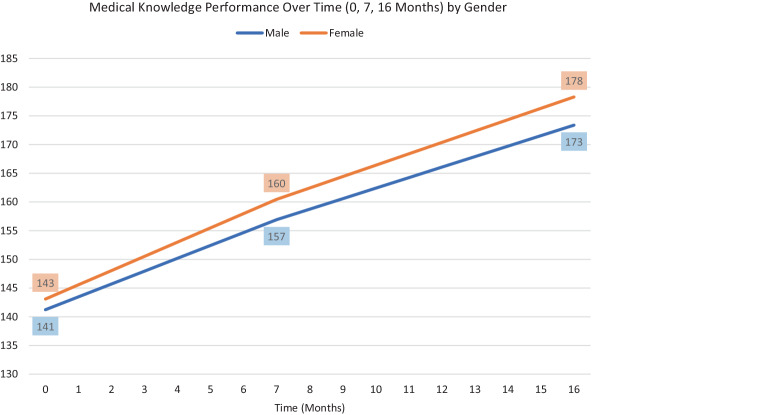
Growth trajectory comparison between male vs. female students during the 16 months

Growth curve modeling is most powerful when explanatory variables (or covariates) are added to explain variability in individual developmental trajectories. There are two types of covariates in growth modeling: a) time-invariant variable (e.g. gender, MCAT score) representing variables with values that do not change over time, and b) time-varying variables (e.g. longitudinal measures of burnout level or amount of feedback received) representing values that change over time. Incorporating these covariates helps to explain and directly evaluate the hypothesis around whether the variables are associated with higher or lower starting point (intercept) and slower or faster change over time (slope) [10]. These types of models are helpful if we want to investigate what factors or interventions have the most impact despite initial differences in an individual learner’s starting point. In the example study, we investigated whether the growth trajectory differed by gender status (Fig. 2) and growth modeling revealed that an initial performance gap actually widened during medical school. As demonstrated, having a theory or hypothesis driven approach to longitudinal study design and analysis yields the most interpretable model and results.

## In summary

- Growth modeling, a data analytic technique for repeated measurements, can be used to investigate change and development over time;
- To optimize the utility of growth modeling, three or more repeated measurements with adequate sample sizes are recommended;
- Report the model fit indices to support the justification of the final model selected;
- Maximize the use of explanatory variables to increase the interpretability and utility of growth models.
